# ASPIS, A Flexible Multispectral System for Airborne Remote Sensing Environmental Applications

**DOI:** 10.3390/s8053240

**Published:** 2008-05-16

**Authors:** Dario Papale, Claudio Belli, Beniamino Gioli, Franco Miglietta, Cesare Ronchi, Francesco Primo Vaccari, Riccardo Valentini

**Affiliations:** 1 DISAFRI, University of Tuscia, via C. de Lellis, 01100 Viterbo Italy; E-mail: darpap@unitus.it; rik@unitus.it; 2 Terrasystem srl, via Pacinotti 5, 01100 Viterbo Italy; E-mail: c.belli@terrasystem.it; 3 CNR-IBIMET, Istituto di Biometeorologia, via Giovanni Caproni 8, 50145 Firenze Italy; E-mail: b.gioli@ibimet.cnr.it; f.miglietta@ibimet.cnr.it; f.vaccari@ibimet.cnr.it.; 4 Barilla G. e R. Fratelli S.p.A., via Mantova 166, 43100 Parma; E-mail: c.ronchi@barilla.it.

**Keywords:** airborne remote sensing, CCD, interferential filters, wheat quality, red edge

## Abstract

Airborne multispectral and hyperspectral remote sensing is a powerful tool for environmental monitoring applications. In this paper we describe a new system (ASPIS) composed by a 4-CCD spectral sensor, a thermal IR camera and a laser altimeter that is mounted on a flexible Sky-Arrow airplane. A test application of the multispectral sensor to estimate durum wheat quality is also presented.

## 1. Introduction

Airborne remote sensing has emerged as an important and powerful tool for environmental monitoring applications, particularly when temporal, spectral or geometric resolutions are important. Despite new high performances satellite sensors are available (e.g. QuickBird, CHRIS, Hyperion, Formsat-2), airborne sensors can still acquire data with geometric resolution that can not be obtained from satellite platforms (in particular temporal and spatial resolutions). The number of sensors commercially available is increasing as hyperspectral instruments are becoming widely used in remote sensing applications, ranging from surface reflectance determination to radiative transfer models inversion [1-4]. These sensors are typically based on 2D line scanners that sample 2D data-lines perpendicular to the flight direction, while the aircraft forward movement drives the shift between two following lines. Such an architecture requires, in order to geo-correct each pixel, a very precise attitude and position determination for each scanning line, and the application of complex software procedures. The resulting geometric resolution and accuracy depends on the accuracy of such navigation data. Typically geometric resolution hardly go below 0.5 – 1 m to avoid the risk of having gaps between adjacent lines. Those characteristics restrict the use of line scanners to specialized groups mainly involved in research activities rather than in commercial application. In addition, this type of sensors requires large aerial platforms to host the instrument and related devices, something that necessarily leads to a substantial cost increase.

Multi spectral systems based on digital photos are less expensive and simpler to use but also less flexible, due to the limited number of acquisition bands and wavelengths that are often fixed and not easily tunable. The minimum bandwidths of those systems are commonly between 10 and 20 nm compared to the 1.5-2 nm available for hyperspectral sensors.

Multispectral sensors based on CCDs technology can acquire an entire image at a determined spectral wavelength on one CCD at a time. Typically 3 or 4 CCDs are used, allowing the recording of 3-4 different spectral bands. The geometric processing of this kind of data requires typically the ortho-projection of the image that can be made manually based on GCP (ground control points) or automatically using aircraft position and attitude data recorded at the time of the image acquisition.

The maximum number of spectral bands and their width restrict the application of such a system compared to hyperspectral sensors but it is also important to consider that the most common vegetation indices are commonly based on few spectral bands [5-7].

The availability of opto-electronic components has increased over the last few years with a series of new high quality and low cost components which are simple to install and use. This is particularly true in the imaging sector with new CCDs and cameras; products with resolutions of more than 6 M-pixels, radiometric resolution of 12-14 bits and very high signal-to-noise ratio are available on the market. Starting from these commercially available components, the Department of Forest Environment and Resources (DISAFRI) of the University of Tuscia designed and built a new sensor, the Advanced Spectroscopic Imaging System (ASPIS) with the aim of obtaining a flexible and simple-to-use instrument, with relatively low maintenance and operational cost. Characteristics that were to limit measurements precision and accuracy compared to other more expensive sensors, but that offer a suitable alternative for a range of airborne remote sensing applications in which very high accuracy is not necessary or at least not for both geometric and radiometric at the same time. The ASPIS system was designed to support and make affordable a large number of application of airborne remote sensing techniques with large potential benefits for the end users.

This paper describes the sensor and illustrates an initial test application. in which the quality of durum wheat grains was based on the prediction of the nitrogen content of the foliage detected by remote sensing. World durum wheat (*Triticum durum* Desf.) production is estimated to be approximately 26 millions tons in 2005/06 [8] and Italy produced in 2005/06, 3.5 millions tons of durum wheat mainly in the agricultural areas of Puglia and Sicilia regions in the southern part of the country. Wheat protein concentration has long been known as a significant determinant of both nutritional [9] and dough qualities. Genetic differences in grain protein concentration among wheat cultivars are considered as intrinsic factors to affect grain protein [10-14]. especially the nitrogen transfer efficiency in the post-anthesis phase [15, 16]. Wheat leaves are the main source of amino acids for grain protein synthesis [16-18]. In the application presented here, vegetation indices were used to assess the nitrogen content of the canopy and the leaves with the basic idea that larger amino acids availability in the foliage may drive an increase in grain protein content.

## 2. The ASPIS system

The ASPIS was designed and built by DISAFRI (University of Tuscia), together with private companies like DTA s.r.l. (Pisa, Italy) and Optec s.p.a. (Milano, Italy) that built respectively the cameras and the lens. ASPIS prototyping was funded by CORIAL (Consorzio Ricerche Alimentari, Barilla Spa) and the Italian Minister of the University and Research with the aim of engineering a flexible and low cost sensor to be used in combination with a crop simulation model developed by the Institute of Biometeorology (IBIMET CNR; Firenze, Italy) that predicts yield and protein contents of durum wheat at the national scale.

**Figure 1.**
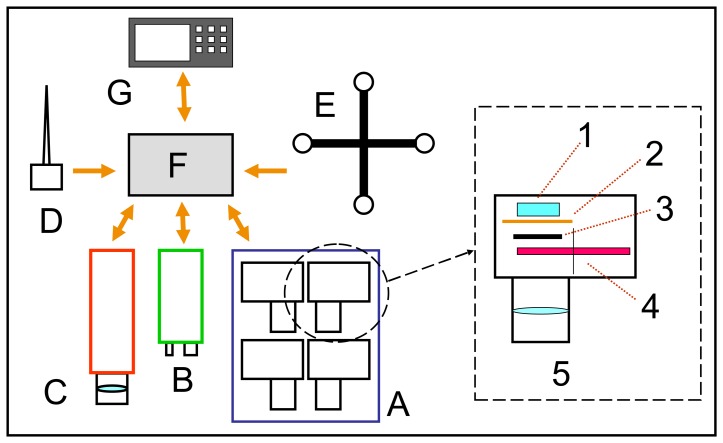
the ASPIS system: A: multispectral sensor, B: laser altimeter, C: thermal IR camera, D: GPS, E: GPS Attitude. All the sensors are connected to the PC (F) and can be controlled by the operator (G); in the dashed square the particular of one camera: 1: Peltier cell, 2: CCD, 3: shutter, 4: wheel with filters, 5: lens.

The main characteristics of the ASPIS system (figure 1) are the simple robust structure, flexibility, low cost of realization and simple use. Flexibility and low cost also guided the choice of the aircraft platform; ASPIS is in fact installed on a SKY ARROW 650 TC, a very small certified single engine aircraft that will be later on in this paper (figure 5). The main component of ASPIS is a multispectral sensor based on 4 CCD cameras that is controlled by a dedicated software (Vision Pro, DTA s.r.l.). The multispectral images are recorded on a compact industrial PC with a 12” LCD flat screen together with all the other sensors that complete the system: a thermal IR camera FLIR SC500 with a CCD 320×240 and spectral sensitivity range from 7.5 μm to 13.0 μm, a laser altimeter Riegl LD90 with a range of about 500 m and a pulse measurement frequency of 100 Hz and two GPS systems.

### 2.1. The multispectral sensor

The multispectral sensor is made of 4 digital camera, each one with a CCD, that acquire images at the same time [19-21], a wheel with eight positions where it is possible to mount the interferential filters, the shutters and the lens, that are connected to the PC through a multiplexer box. Data acquired by the GPS systems are also registered to the PC. The whole system, weights about 40 kg, is directly powered by a dedicated aircraft auxiliary alternator, while a small battery ensures power supply during short time power deficits. All the instruments power lines are stabilized and filtered.

Each camera is made by a CCD Kodak KAF400e. This is a half inch Full Frame Transfer CCD with 768 x 512 pixels with a pixel dimension of 9 x 9 microns sensible in the spectral range 375 – 1000 nm. The radiometric resolution is 14 bits and the CCDs are cooled using Peltier cells to reduce the noise.

There are two electro-mechanic shutters synchronized to obtain exposure time of about 1/1000 sec. compared with the minimum exposure time of the single shutter that is about 1/500 sec. The electronic shutter, faster then the electro-mechanic, were rejected because when the system was in development they had a very low transmission efficiency.

**Figure 3.**
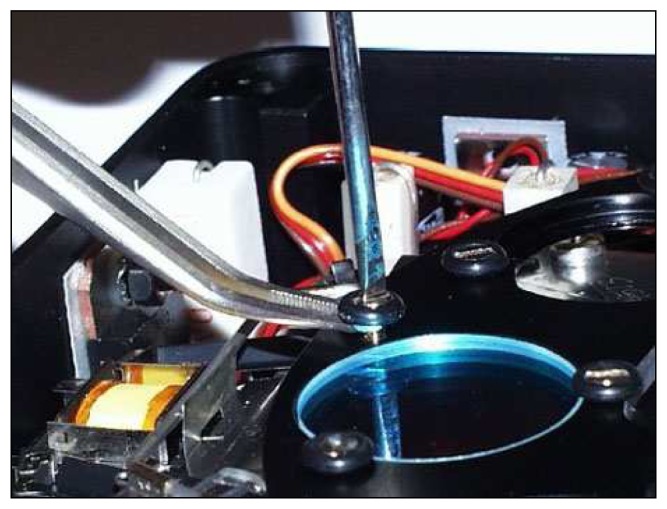
Spectral filter mounted on the 8 positions wheel.

The interferential filters, distributed by MiCos Italia GmbH (http://www.micos.it), are mounted on a wheel with eight different positions (figure 3) that are user selectable during the flight through the control software. The time interval to complete the filter positioning is 0.05 sec. The same software allows setting other camera parameters such as exposure time independently for each of the four channels, CCD gain and temperature of the Peltier's cells.

The ASPIS's lenses system is made of 4 *retrofocus* lenses manufactured to have as the smallest distortion (15 cm length and 7 cm diameter) and robust enough to tolerate vibrations due to the aircraft, a FOV of 38° (focal length of 12.5 mm) and a maximum diaphragm aperture of f/2.

#### 2.1.1. Sensor calibration

**Figure 4.**
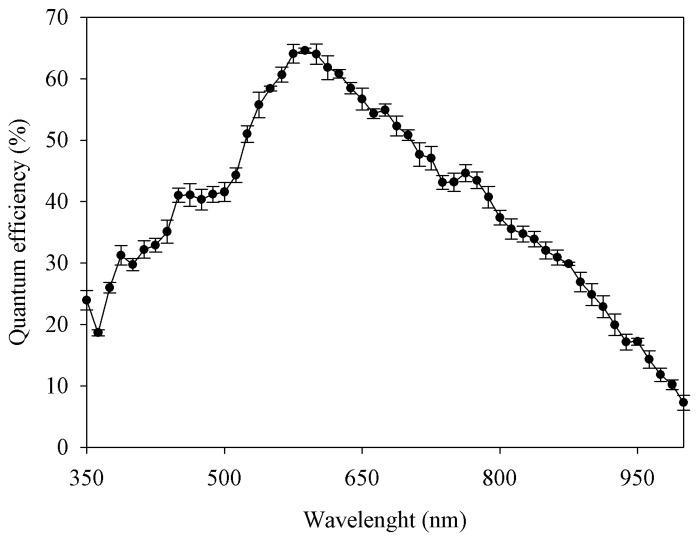
Measured quantum efficiency of the 4 CCD (average value and standard deviation).

Different calibrations procedures were applied on the system, concerning in particular the CCD, the filters and the optics. Quantum efficiency of the CCDs were measured using a spectrometer with a wavelength step of 12.5 nm. The results are shown in figure 4. Filters' transmittance was also measured using a spectrometer and the results varied between 65% and 85%. All the measured characteristics of filters transmittance and CCD efficiency were used in the radiometric correction.

Another important set of tests was made on the optics to verify lens quality, quantum efficiency and geometric distortion. In particular, the Modulation Transfer Function and the Mean Radial Distortion Curve have shown good lens quality and the resulting equations are used in the geometric correction of the images. Also the Spectral Transmittance Curve had a very low level of interference with transmittance between 89.5 and 95% for the different wavelengths.

The ASPIS sensor is not yet radiometrically calibrated. It means that is not possible to obtain the radiance to the sensor starting from the Digital Number using a general transformation function derived experimentally. In the applications where radiance value is necessary to derive reflectance calculations, targets on the ground are to be used.

### 2.2. GPS Attitude

Two GPS-based systems are installed onboard of the aircraft. The first (mod DG14, Astech, USA) is a single frequency GPS unit that is used to acquire aircraft position and as synchronization source for the cameras. Trigger signals generated by the GPS at a programmable frame rate (from 1Hz to 0.1 Hz) are used to start the acquisition of each frame, so that very precise coupling between sensor position and corresponding acquired image can be achieved. The second (mod ADU3, Astech, USA) is a 4 antennas vectorial system used to measure platform attitude angles (pitch, roll and heading). The antennas are mounted on the wings, on the tail and on the front of the aircraft to form an array. Attitude angles are logged at 1Hz frequency, and the synchronization with the acquired images is ensured by matching during post-processing satellite clock information provided by the two GPS. The accuracy of these measurements is ± 3.0 m for 3D position without differential correction, ± 0.12° (pitch and roll angles) ± 0.06° (heading angle).

### 2.3. The platform

**Figure 5.**
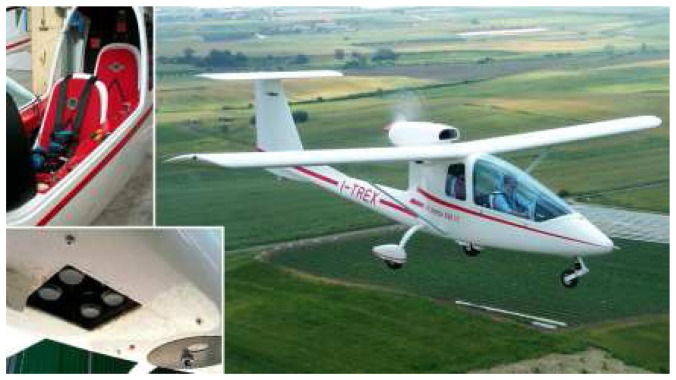
The platform: Sky Arrow 650TC.

The ASPIS prototype system was designed for a Sky Arrow 650TC aircraft (figure 5), built by Iniziative Industriali Italiane SpA (Rome, Italy). This aircraft, has two seats in line, high wings, a 1300 cc Rotax 912S engine that allows to fly with a cruise speed between 70 and 100 knots, with a range of about 3.5 hours. The airplane has a very easy and flexible handling, it can take off and land from relatively short grass runways, and can perform operational flights between 100 and 4000 meter asl. The Sky Arrow was selected because it meets some important requirements needed to install remote sensing instrumentation onboard: it has two downward looking openings, the first with a rectangular shape of 215 x 235 mm, the second with a circular shape of 360 mm diameter, that allows the mounting of multiple optic systems. In addition it has mounting plates to host all the other associated instruments and electronics and it is equipped with two 12 VDC 20A power lines dedicated only to the external equipment. The ASPIS sensor has been mounted on a shock absorber steel plate on the rectangular trap door.

### 2.4. Deployment

The prototype described is capable of acquiring images in 4 different spectral bands selectable also during the flight, with a sub-metric spatial resolution and good radiometric resolution. The images have an overlap of about 2/3, characteristic that makes possible the use of the data acquired for stereo-view and Digital Elevation Models generation.

Spatial resolution of the images is a function of the flight altitude. Depend on the aircraft altitude range, the nadir pixel size varies between few centimetres to few meters. Examples of pixel sizes and image dimensions as function of the flight height are presented, for normal operative flight conditions, in Table 1.

## 3. Application

In this paper we present an application of the ASPIS system that was made to assess the protein content of durum wheat grains before harvest using remotely sensed information. Nitrogen and protein contents are indeed an important parameter to assess the durum wheat quality and the final goal of the project was to test the applicability of remote sensing techniques to have an assessment of the durum wheat quality at regional scale using reference calibration fields.

The basic assumption was that the protein content of the grains at maturity is intimately related to nitrogen content of the upper canopy leaves [22-24], i.e. with their chlorophyll content [2, 25]. And chlorophyll content is a parameter that can be directly assessed with the spectral reflectance in the red and near infrared regions of the spectrum [26, 27].

The experimental activity has been divided in two phases: at first, an analysis of the spectral reflectance using a portable spectrometer was made on different wheat fields with different wheat cultivars and nitrogen fertilization levels; then, more vegetation indices were compared to choose the ideal 4 spectral bands combinations to be use with the ASPIS sensor for the acquisition of images of the calibration fields.

### 3.1. The calibration fields

**Figure 6.**
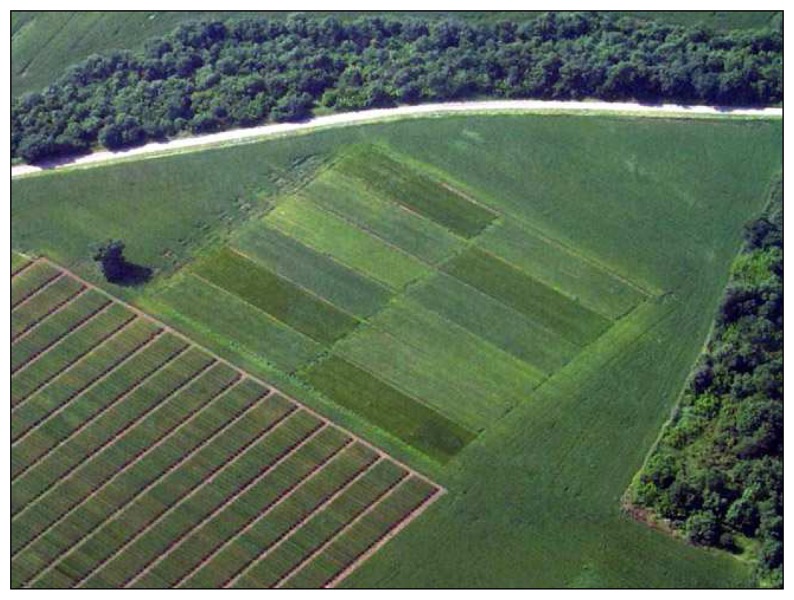
The Cerignola calibration field.

Three calibration fields were created in the Puglia (south Italy) region (Foggia, Chieuti and Cerignola municipalities). Field plots of 30 × 30 meters were divided in 12 sub-plots (15 × 5 meters) (figure 6) in which 3 different durum wheat cultivars (*Svevo*, *Simeto* and *Colosseo*) were grown at 4 different nitrogen fertilization levels.: 0, 40, 80 and 120 Kg N ha^-1^. The three calibration fields were used in 2002 to select the 4 spectral band combination to be later on used with the airborne sensor; flights were then made in 2003 over the field plots in Cerignola.

### 3.2 Ground measurements

A handheld GER 3700 portable spectrometer was used to acquire wheat spectral signatures between 350 and 2500 nm. Four measurements were made for each sub-plot and plant samples were harvested immediately after the measurements. Due to the field of view of the instrument (15 degree) and the measurement height (2 meters) the area investigated was 0.5 x 0.5 meters for each measurement point and the plants were sampled within these areas.

Sampled material was then dried in an oven and 10 sub-sample were analyzed using a Near Infra Red analyzer (FOSS NIR SYSTEM 6500) to measure the nitrogen content of the leaves [28]. The average of the ten measurements was finally used to determine the mean protein value content (% of dried matter) of each sample.

The measurements were made at different dates in April and leaf nitrogen content increased over time up to a maximum reached at the end of the ear emergence stage [29] to be then translocated towards the grains, during grain filling.

In order to investigate and choose the most appropriate vegetation index, only wavelengths that could be possibly acquired by the ASPIS sensor were considered; in this way, the range was restricted to visible and NIR up to 850 nm, where the CCD efficiency is 30%. A comparison between different vegetation indices and the plants N content is shown in Table 2.

Generally, the normalized indices (NDVI and SIPI, less evident for the NPCI) had saturation problems, as already reported elsewhere [33-36]. We found better relationships between leaf N content and spectral indices using the PSR and other indices in the red-edge area. In particular with the last two which are reported in Table 2, which are both based on the shape or slope of the reflectance curve between 670 and 770 nm [37]. Those indices are in fact very sensitive to LAI and chlorophyll concentration while they minimize the spectral noise due to soil background, atmosphere and solar zenith angle [38-40]. For such reasons we decided to use a red-edge based index with the airborne sensor.

We processed the ground spectral data in order to determine spectral bands combinations and associated bandwidths that could be used on ASPIS. Due to the system characteristics (only 4 acquisition bands with limited narrow bandwidth) the “flex point wavelength” could not be used, as part of the necessary information was lost when averaging ground spectral data over broader bands in the red edge region. Also the area under the curve was discarded because the system was not yet radiometrically calibrated. [41]. Instead a better vegetation index in the red-edge slope was selected, as the mean red-edge slope that was efficiently measurable with the four broad bands.

The fine tuning of the 4 wavelengths that best describe the proposed red-edge index maintaining most of the information, resulted in 4 interferential filters being order, with the characteristics reported in table 3.

**Figure 7.**
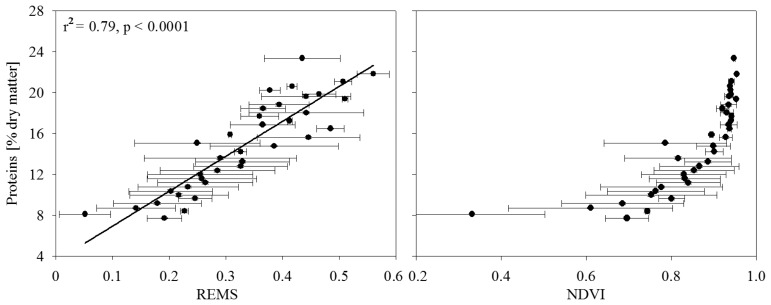
Relation between the plant protein content and two vegetation indexes: REMS (left side) and NDVI (right side). Data from two different calibration fields, three wheat cultivars and four fertilization levels have been grouped into equal protein classes, thus only horizontal bar is reported, as the standard deviation of the measurements falling in each protein class. Leaf protein content was calculated on the basis of nitrogen concentration determinations made by NIR.

The formulation of the red edge slope index was consequently:

(1) $$\text{REMS}=\frac{\partial \rho }{\partial \lambda }≅\frac{1}{\text{n}}\sum_{\text{i}=1}^{4}\frac{\rho_{\text{i}+1}-\rho_{\text{i}}}{\lambda_{\text{i}+1}-\lambda_{\text{i}}}$$

where REMS = Red Edge Mean Slope, ρ_i_ and λ_i_ are reflectance and wavelength for the band i and n is the number of spectral bands used (4 in this case). Figure 7 (left side) shows the relationship between the REMS index and the leaf nitrogen content measured on the basis of the ground measurements described above. Horizontal bars account there not only for the uncertainty of the estimation, but also for variability in the response of the wheat cultivars, fertilization levels, and also soil conditions of the experimental plots planted in different areas. Despite such heterogeneity the proposed index was still able to explain 79% of the variance, thus resulting to be a potentially very good predictor of leaf nitrogen content in ‘real world’ conditions. The relationship between NDVI and protein content is shown in the right panel of figure 7, where the saturation shape that makes NDVI not a suitable index for this study, is clearly detectable.

### 3.2. Airborne measurements

The flight over the Cerignola calibration was made on April 14^th^ 2003, at the beginning of the ear emergence stage.

The overflight was made under clear sky conditions, at 600 meter above ground with a resultant pixel geometric resolution of about 0.5 meters. During the flight, 10 plants samples were taken from each sub-plot and leaf nitrogen content was determined as described previously. In addition, the grain protein content was measured also after harvest

**Figure 8.**
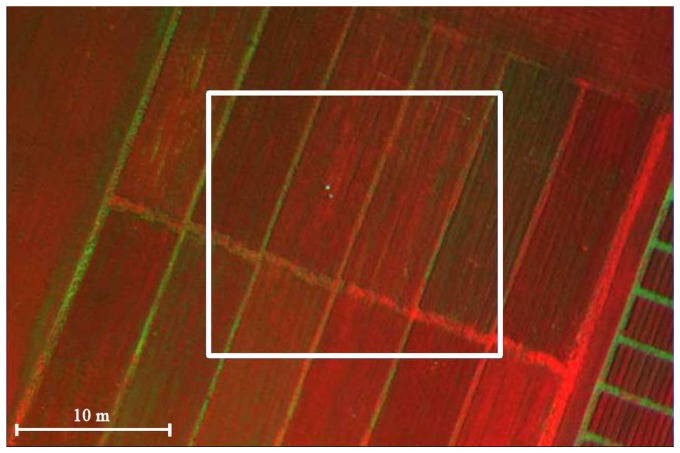
Example of ASPIS calibration filed false colours composition image. The white square indicates the part of the images used in the index calculation to reduce the bidirectional reflectance effect.

The REMS index was calculated for each of the 12 sub-plots on the basis of a total of 10 images. To limit the bidirectional reflectance effect to the index calculation, only the central part of each image was used to calculate REMS, with a FOV of about 8 degree around the nadir axis (figure 8). In each of the 10 images there were always 2 or more sub-plots falling in the sub-area considered (white square in figure 8). For each sub-plot, all the pixels available (commonly between 700 and 12000 in each frame) in the whole set of images have been used to calculate the mean REMS index value.

### 3.3. Results and Discussion

**Figure 9.**
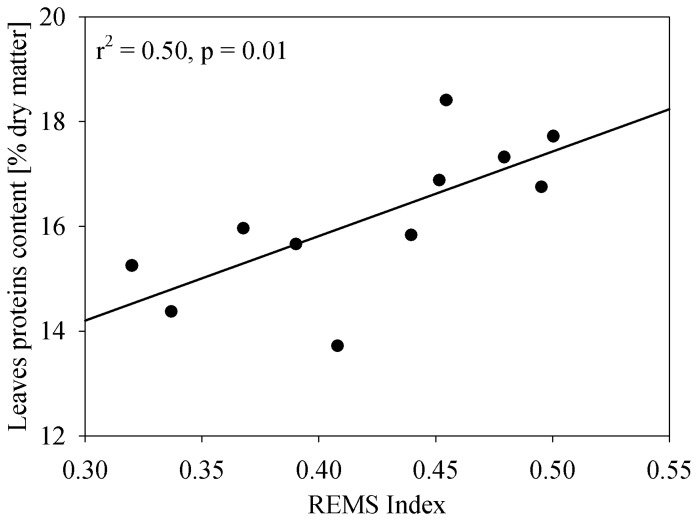
Relation between REMS index calculated using the ASPIS images and the plants protein content for the different sup-fields

The correlation between the REMS index calculated using the ASPIS images and the leaf protein content is shown in figure 9. Also in this case, leaf nitrogen was converted into protein using a simple multiplying factor [28]. The vegetation index explains 50% of the protein content variance but the result is based on 11 and not 12 sub-plots since protein content determination in one individual plot was not reliable. Such error occurred in one sub-plot was also detected in the subsequent comparison between leaf and grain protein content; the residuals in the protein content prediction have a standard deviation of about 0.73 while the residual for the sub-plot with problems is 2.4 times larger than three time the standard deviation (not shown here).

**Figure 10.**
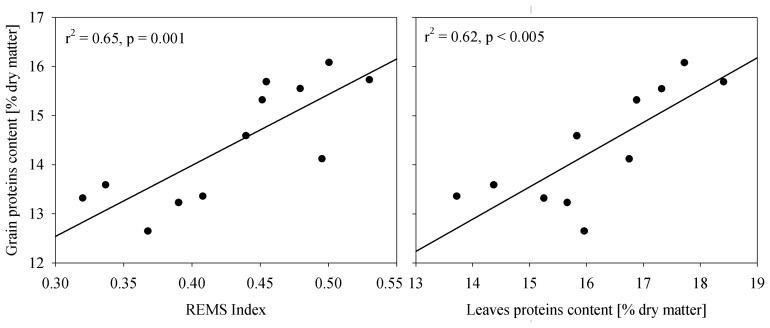
Relations between grain protein content measured after yield and REMS from ASPIS (left side) and plant protein content (right side).

The relationship between wheat grain protein content measured after harvest and REMS index is shown in the left panel of figure 10. Better correlation compared to that reported in figure 9 is likely due to a lower uncertainty in the protein content determination (protein content determination after harvest is more representative of the spatial heterogeneity). It is also interesting to observe that the correlation between post-harvest grain protein content and remotely sensed data is slightly higher and significant than the correlation with the plant protein content measured with samples (r^2^ 0.65 *vs* 0.62, p=0.001 *vs* p<0.005, figure 10 right side).

This result is interesting because it suggests that it is possible to obtain a first assessment of durum wheat protein content well before harvest, with at least the same accuracy that can be obtained with the classic filed sampling method, but with the obvious advantages of the remote sensing techniques, like, for instance, the homogeneous applicability to large regions and the cost effectiveness. This is also confirmed by other studies that, although difficult to compare directly with this application, have demonstrated that spectral reflectance measurements can be used effectively to predict grain yield and protein content [42-44].

It is important to highlight here that the application which has been illustrated is just a first example that need further analysis and refinements, although the results obtained are interesting and promising. For instance, new indices should be considered and added to the analysis, like the VIs based on the green and NIR wavelength that are closely related to the chlorophyll content since those consider also the greenness of the canopy [41, 45]. At the same time, once the sensor is radiometrically calibrated, it will be important to test the correlation between airborne vegetation index and protein content flying over different calibration fields in the region. Despite those limitations, this application is a good example of operational uses of airborne remote sensing in an area where this technique is still not popular and where there is an obvious economic advantage compared to the traditional field survey methods.

## 4. Conclusions

In this paper a new airborne multispectral sensor has been described. The main characteristics of the ASPIS system are the flexibility and the low construction and operational costs. Together with the sensor description a first application has been presented, too. Although this is a preliminary test and further developments are clearly required needed, it is a classical example of an application field where airborne remote sensing can be a very useful operational tool, if economically sustainable and a relatively simple data processing protocol.

Disadvantage of airborne remote sensing, with respect to satellite remote sensing, are commonly the additional geometric correction needed due to aircraft movement correction, and the number of images to be processed is much higher respect to satellites for the same areas. However there are applications in which high accuracy is not required and where sensors like ASPIS could represent a good compromise between cost and quality. The application of ASPIS to estimate the wheat protein content has been illustrated in detail, and empirical relationships have been considered to account for the variability due to different cultivars, different fertilization strategies and soils, thus potentially allowing the application of this method at the regional scale to produce maps of the wheat quality.

Airborne remote sensing is becoming more and more popular and used and in this special issue another sensor is presented [46]. This hyperspectral sensor (AVIS-2) is somehow complementary to the one presented in this paper: in fact, both the sensors are useable with simple and flexible platforms, but while the AVIS has higher spectral resolution (64 bands with bandwidth of 7.3 nm), the ASPIS has higher geometric resolution (since limited only by the flight altitude) and gives the possibility to obtain stereo images with 80% overlap that can be used to derive surface elevation models.

The ASPIS system has been used also in other applications like the wildfire burned area mapping in the Lazio region [47] and the mapping of chestnut phyto-pathologies in central Italy [48] that have been useful also to understand the main problems of the sensor and possible improvements. For example, the 4 independent lenses (practically 4 independent cameras with in theory parallel view axis) can lead to problems in the spectral images overlapping since, although very small, divergences or convergences of the 4 view axis are inevitable and additional processing then is required. For this reason a new version of the sensor is currently under development where only one lens and a beam splitters system will be used. Also the CCD will be replaced with more efficient models that have become available to compensate the loose of light given by the beam splitter.

## Figures and Tables

**Figure 2.**
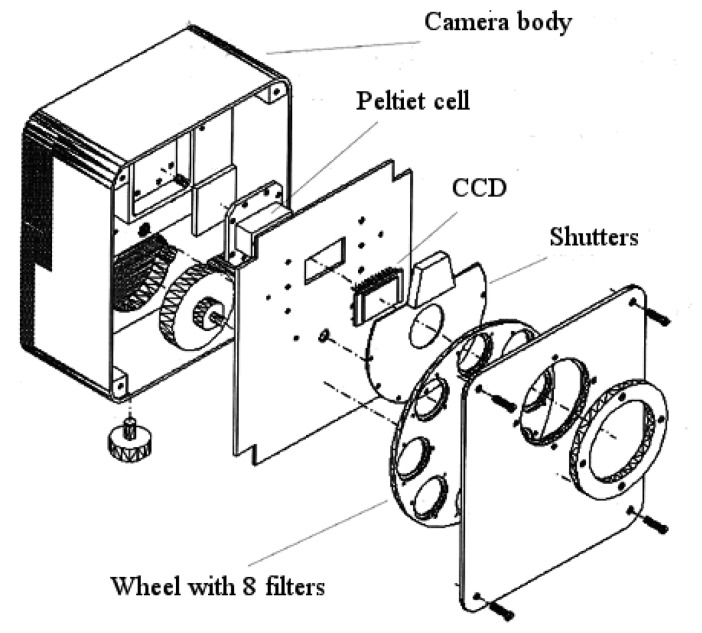
one of the ASPIS sensor's cameras.

**Table 1.** Nadir pixel sizes and image dimensions at ground as function of the flight height.

**Flight height (m)**	**Nadir pixel size (m)**	**Image size (m)**
200	0.15	115 × 77
500	0.37	288 × 192
1000	0.75	575 × 383
1300	0.97	747 × 498
1500	1.12	862 × 575
2000	1.5	1150 × 767

**Table 2.** Vegetation indexes calculated [30-32].

**Vegetation index**	**Calculation**
NDVI (Normalized Difference Vegetation	(R780-R680)/(R780+R680)
NPCI (Normalized difference pigment index)	(R430-R680)/(R430+R680)
PSR (Pigment simple ratio)	R430/R680
SIPI	(R445-R800)/(R445+R800)
Q [Area dR_re_ 680-780]	area under the curve between 680 and 780
nm [Length_max λ_re_]	Red-edge flex point wavelength
maxder [max dR_re_]	Red-edge maximum derivative value

**Table 3.** Characteristics of the interferential filters used.

**Central wavelength (nm)**	**Transmittance (%)**	**FWHM Δλ_0.5_** **(nm)**	**Bandwidth 10% of** **T_max_ (nm)**
701.0	83	7.8	13.0
719.3	80	9.5	17.3
733.5	72	9.2	15.0
748.5	83	8.9	15.7

## References

[1] Ganapol B.D., Johnson L.F., Hammer P.D., Hlavka C.A., Peterson D.L. (1998). LEAFMOD: a new within-leaf radiative trensfer model. Remote Sensing of Environment.

[2] Jacquemoud S., Ustin S.L., Verdebout J., Schmuck G., Andreoli G., Hosgood B. (1996). Estimating Leaf Biochemistry Using the PROSPECT Leaf Optical Properties Model. Remote Sensing of Environment.

[3] Meroni M., Colombo R., Panigada C. (2004). Inversion of a radiative transfer model with hyperspectral observations for LAI mapping in poplar plantations. Remote Sensing of Environment.

[4] Renzullo L.J., Blanchfield A.L., Guillermin R., Powell K.S., Held A.A. (2006). Comparison of PROSPECT and HPLC estimates of leaf chlorophyll contents in a grapevine stress study. International Journal of Remote Sensing.

[5] Dash J., Curran P.J. (2004). The MERIS terrestrial chlorophyll index. International Journal of Remote Sensing.

[6] Gitelson A.A., Vina A., Ciganda V., Rundquist D.C. (2005). Remote estimation of canopy chlorophyll content in crops. Geophysical Research Letters.

[7] Thenkabail P.S., Smith R.B., De Pauw E. (2002). Evaluation of Narrowband and Broadband Vegetation Indeces for Determining Optimal Hyperspectral Wavebands for Agricultural Crop Characterization. Photogrammetric Engineering & Remote Sensing.

[8] USDA (2006). Production Estimates and Crop Assessment.

[9] Gunthardt H., McGinnis J. (1957). Effect of Nitrogen Fertilization on Amino Acids in Whole Wheat. Journal of Nutrition.

[10] Bhatia C.R. (1975). Criteria for early generation selection in wheat breeding programmes for improving protein productivity. Euphytica.

[11] Jamieson P.D., Zyskowski R.F., Semenov M.A. Modelling genetic variability in wheat quality.

[12] Johnson V.A., Mattern P.J., Peterson C.J., Kuhr S. (1985). Improvement of wheat protein by traditional breeding and genetic techinques. Cereal Chemistry.

[13] Kramer T. (1979). Environmental and genetic variation for protein content in winter wheat (Triticum aestivum L.). Euphytica.

[14] Rostami M.A., O'Brien L. (1996). Differences among bread wheat genotypes for tissue nitrogen content and their relationship to grain yield and protein content. Australian Journal of Agricultural Research.

[15] Barbottin A., Lecomte C., Bouchard C., Jeuffroy M.-H. (2005). Nitrogen Remobilization during Grain Filling in Wheat: Genotypic and Environmental Effects. Crop Science.

[16] Jenner C.F., Ugalde T.D., Aspinall D. (1991). The Physiology of Starch and Protein Deposition in the Endosperm of Wheat. Functional Plant Biology.

[17] Fernandez-Figares I., Marinetto J., Royo C., Ramos J.M., Garcia del Moral L.F. (2000). Amino-Acid Composition and Protein and Carbohydrate Accumulation in the Grain of Triticale Grown under Terminal Water Stress Simulated by a Senescing Agent. Journal of Cereal Science.

[18] Garcia del Moral L.F., Boujenna A., Yanez J.A., Ramos J.M. (1995). Forage Production, Grain Yield, and Protein Content in Dual-Purpose Triticale Grown for Both Grain and Forage. Agronomy Journal.

[19] Metternicht G. (2003). Vegetation indices derived from high-resolution airborne videography for precision crop management. International Journal of Remote Sensing.

[20] Edirisinghe A., Louis J.P., Chapman G.E. (1999). Radiometric Calibration of Multispectral Airborne Video Systems. International Journal of Remote Sensing.

[21] Edirisinghe A., Chapman G.E., Louis J.P. (2001). A simplified method for retrieval of ground level reflectance of targets from airborne video imagery. International Journal of Remote Sensing.

[22] Boman R.K., Westerman R.L., Raun W.R., Jojola M.E. (1995). Time of Nitrogen Application: Effects on Winter Wheat and Residual Soil Nitrate. Soil Science Society of America Journal.

[23] Scheromm P., Martin G., Bergoin A., Autran J.C. (1992). Influence of nitrogen fertilizer on the potential bread-baking quality of two wheat cultivars differing in their responses to increasing nitrogen supplies. Cereal Chemistry.

[24] Woodard H.J., Bly A. (1998). Relationship of nitrogen management to winter wheat yield and grain protein in South Dakota. Journal of Plant Nutrition.

[25] Blackmer T.M., Schepers J.S., Varvel G.E. (1994). Light Reflectance Compared with Other Nitrogen Stress Measurements in Corn Leaves. Agronomy Journal.

[26] Daughtry C.S.T., Walthall C.L., Kim M.S., de Colstoun E.B., McMurtrey J.E. (2000). Estimating Corn Leaf Chlorophyll Concentration from Leaf and Canopy Reflectance. Remote Sensing of Environment.

[27] Gamon J.A., Field C.B., Joel G., Goulden M.L., Griffin K.L., Hartley A.E., Geeske J., Penuelas J., Valentini R. (1995). Relationships between NDVI, canopy structure and photosynthetic activity in three Californian vegetation types. Ecological Applications.

[28] Jones D.B. (1931). Factors for converting percentages of nitrogen in foods and feeds into percentages of protein. USDA Circular.

[29] Filella I., Serrano L., Serra J., Peñuelas J. (1995). Evaluating Wheat Nitrogen Status with Canopy Reflectance Indices and Discriminant Analysis. Crop Science.

[30] Zarco-Tejada P.J., Pushnik J.C., Dobrowski S., Ustin S.L. (2003). Steady-state chlorophyll *a* fluorescence detection from canopy derivative reflectance and *double-peak* red-edge effects. Remote Sensing of Environment.

[31] Elvidge C.D., Zhikang C. (1995). Comparison of Broad-Band and Narrow-Band Red and Near-Infrared Vegetation Indices. Remote Sensing of Environment.

[32] Read J.J., Tarpley L., McKinion J.M., Reddy K.R. (2002). Narrow-Waveband Reflectance Ratios for Remote Estimation of Nitrogen Status in Cotton. J Environ Qual.

[33] Clay D.E., Kim K.-I., Chang J., Clay S.A., Dalsted K. (2006). Characterizing Water and Nitrogen Stress in Corn Using Remote Sensing. Agronomy Journal.

[34] Danson F.M., Plummer S.E. (1995). Red-edge response to forest leaf area index. International Journal of Remote Sensing.

[35] Jackson T.J., Chen D., Cosh M., Li F., Anderson M., Walthall C., Doriaswamy P., Hunt E.R. (2004). Vegetation water content mapping using Landsat data derived normalized difference water index for corn and soybeans. Remote Sensing of Environment.

[36] Eitel J.U.H., Long D.S., Gessler P.E., Smith A.M.S. (2007). Using in-situ measurements to evaluate the new RapidEye (TM) satellite series for prediction of wheat nitrogen status. International Journal of Remote Sensing.

[37] Patel N.K., Patnaik C., Dutta S., Shekh A.M., Dave A.J. (2001). Study of crop growth parameters using Airborne Imaging Spectrometer data. International Journal of Remote Sensing.

[38] Broge N.H., Mortensen J.V. (2002). Deriving green crop area index and canopy chlorophyll density of winter wheat from spectral reflectance data. Remote Sensing of Environment.

[39] Filella I., Peñuelas J. (1994). The red edge position and shape as indicators of plant chlorophyll content, biomass and hydric status. International Journal of Remote Sensing.

[40] Mauser W., Bach H., Hill J, Megier J (1995). Imaging spectroscopy in hydrology and agriculture?determination of model parameters. Imaging spectrometry: a tool for environmental observations..

[41] Gitelson A.A., Viña A., Verma S.B., Rundquist D.C., Arkebauer T.J., Keydan G., Leavitt B., Ciganda V., Burba G.G., Suyker A.E. (2006). Relationship between gross primary production and chlorophyll content in crops: Implications for the synoptic monitoring of vegetation productivity. Journal of Geophysical Research.

[42] Hansen P.M., Jørgensen J.R., Thomsen A. (2002). Predicting grain yield and protein content in winter wheat and spring barley using repeated canopy reflectance measurements and partial least squares regression. Journal of Agricultural Science (Cambridge).

[43] Wenjiang H., Jihua W., Zhijie W., Jiang Z., Liangyun L., Jindi W. (2004). Inversion of foliar biochemical parameters at various physiological stages and grain quality indicators of winter wheat with canopy reflectance. International Journal of Remote Sensing.

[44] Xue L.-H., Cao W.-X., Yang L.-Z. (2007). Predicting Grain Yield and Protein Content in Winter Wheat at Different N Supply Levels Using Canopy Reflectance Spectra. Pedosphere.

[45] Gitelson A.A., Keydan G.P., Merzlyak M.N. (2006). Three-band model for noninvasive estimation of chlorophyll, carotenoids, and anthocyanin contents in higher plant leaves. Geophysical Research Letters.

[46] Oppelt N., Mauser W. (2007). Airborne Visible / Infrared Imaging Spectrometer AVIS: Design, Characterization and Calibration. Sensors.

[47] Carlini M., Valentini R., Belli C., Capitoni B., Papale D. (2006). Progetto SIMIB: mappe degli incendi e valutazione dei danni da oggi a portata di mouse. Silvae.

[48] Vannini A., Vettraino M., Fabi A., Montaghi A., Valentini R., Belli C. (2005). Monitoring Ink Disease of Chestnut with the Airborne Multispectral System A.S.P.I.S. Acta Horticulturae.

